# Disulfiram combined with copper inhibits metastasis and epithelial–mesenchymal transition in hepatocellular carcinoma through the NF‐κB and TGF‐β pathways

**DOI:** 10.1111/jcmm.13334

**Published:** 2017-11-17

**Authors:** Yi Li, Li‐Hui Wang, Hao‐Tian Zhang, Ya‐Ting Wang, Shuai Liu, Wen‐Long Zhou, Xiang‐Zhong Yuan, Tian‐Yang Li, Chun‐Fu Wu, Jing‐Yu Yang

**Affiliations:** ^1^ Department of Pharmacology Shenyang Pharmaceutical University Shenyang China; ^2^ Benxi Institute of Pharmaceutical Research Shenyang Pharmaceutical University Shenyang China

**Keywords:** disulfiram, copper, metastasis, EMT, TGF‐β

## Abstract

Late‐stage hepatocellular carcinoma (HCC) usually has a low survival rate because of the high risk of metastases and the lack of an effective cure. Disulfiram (DSF) has copper (Cu)‐dependent anticancer properties *in vitro* and *in vivo*. The present work aims to explore the anti‐metastasis effects and molecular mechanisms of DSF/Cu on HCC cells both *in vitro* and *in vivo*. The results showed that DSF inhibited the proliferation, migration and invasion of HCC cells. Cu improved the anti‐metastatic activity of DSF, while Cu alone had no effect. Furthermore, DSF/Cu inhibited both NF‐κB and TGF‐β signalling, including the nuclear translocation of NF‐κB subunits and the expression of Smad4, leading to down‐regulation of Snail and Slug, which contributed to phenotype epithelial–mesenchymal transition (EMT). Finally, DSF/Cu inhibited the lung metastasis of Hep3B cells not only in a subcutaneous tumour model but also in an orthotopic liver metastasis assay. These results indicated that DSF/Cu suppressed the metastasis and EMT of hepatic carcinoma through NF‐κB and TGF‐β signalling. Our study indicates the potential of DSF/Cu for therapeutic use.

## Introduction

HCC is characterized by an increasing incidence and a poor prognosis 1. Due to the highly vascular nature of the liver, HCC is prone to both intrahepatic and extrahepatic metastases, which are the main cause of treatment failure. Conventional anticancer drugs such as doxorubicin, cisplatin and 5‐fluorouracil show limited efficacy 2. It has been recognized that EMT plays a pivotal role in the progression and metastasis of HCC 3. There is accumulating evidence for an important link between EMT and the invasion, metastasis and self‐renewal traits of cancer cells. Among all the known factors involved in EMT, the transforming growth factor‐beta 1 (TGF‐β) signalling pathways have taken a centre stage 3. TGF‐β signalling plays crucial roles in regulating malignancy initiation, progression and metastasis, including hepatocellular carcinoma 4. The nuclear factor kappa B (NF‐κB) pathway has also been implicated in EMT and metastasis by transcriptionally up‐regulating master‐switch transcription factors required for EMT, such as Slug 5. Coordinated activation of NF‐κB and TGF‐β signalling cascades effectively induces EMT and the expression of genes related to stemness and cell invasion 6.

DSF is an aldehyde dehydrogenase inhibitor that was used as a vermicide in the 1930s 7 and for alcohol aversion therapy in the 1940s 8. The anticancer activity of DSF was reported as early as 40 years ago 9, 10, since when DSF has been widely researched due to its relatively good safety profile and reasonable financial cost. As a result of these studies, DSF has been reported to regulate tumour growth through the inhibition of proteasome activity 11, 12, induction of apoptosis 13, 14, 15, blockage of drug resistance 13, 16, 17, 18, 19, inhibition of invasion and angiogenesis 20, 21, 22 and suppression of stem cell‐like properties 23. Moreover, we found that DSF is highly toxic to cancer cells in a Cu‐dependent manner 24 and has improved anti‐angiogenic activity when combined with Cu. In the field of metastasis, previous studies revealed that DSF inhibited invasion in both tumour and endothelial cells at non‐toxic concentrations through the inhibition of MMP‐2 and MMP‐9 activity 20, 21. It was also found that DSF inhibited TGF‐β‐induced EMT in breast cancer cells in a dose‐dependent manner and inhibited EMT‐associated stem‐like features, migration and invasion of tumour cells, and tumour growth in a xenograft model 25. Although previous research revealed that DSF showed improved cytotoxicity when HepG2 cells were pre‐treated with Cu 26, the anti‐metastatic activity and the molecular anti‐metastatic mechanisms of DSF/Cu in HCC remained unclear. The aim of the current research is to explore these mechanisms.

In this study, we discovered that DSF/Cu showed improved anti‐metastatic activity in HCC compared with DSF alone both *in vivo* and *in vitro*. Down‐regulation of the TGF‐β and NF‐κB pathways contributed to the inhibition of EMT and might be the cause of the improved anti‐metastatic activity of DSF/Cu in HCC. Our results provide supporting evidence that DSF/Cu may be further exploited for therapeutic use.

## Materials and methods

### Reagents

Disulfiram, copper (II) D‐gluconate, calcein‐AM MTT (3‐(4, 5‐dimethylthiazol‐2‐yl)‐2, 5‐diphenyl tetrazolium bromide) and PDTC (pyrrolidine dithiocarbamate) were purchased from Sigma‐Aldrich (St. Louis, MO, USA). The disulfiram‐loaded lipid emulsion was provided by Prof X Tang (Shenyang Pharmaceutical University, China). The drug‐loading content of the disulfiram‐loaded lipid emulsion was 3 mg/ml. The cumulative release of DSF from the lipid emulsion in 120 hrs was more than 60%. The pharmacokinetic properties of DSF in rat plasma after intravenous administration of a dose of 36 mg/kg have been investigated (t_1/2 _= 0.1 hrs and t_1/2d _= 0.3 hrs). A comparison of the biological activity of the disulfiram‐loaded lipid emulsion and conventional DSF has been published 27. Matrigel was purchased from BD Biosciences (San Jose, CA, USA). The primary antibodies against E‐cadherin, Vimentin, Slug and Smad4 were purchased from Cell Signaling Technology (Danvers, MA, USA); antibodies to Ki67, β‐actin, p65 and p50 were obtained from Santa Cruz Biotechnology (Santa Cruz, CA, USA); antibodies to N‐cadherin, Snail+Slug and MMP2 were obtained from abcam (Cambridge, MA, USA).

### Cell culture and cell survival assay

The human hepatocellular carcinoma (HCC) cell lines Hep3B and HepG2 were obtained from the American Type Culture Collection (Manassas, VA, USA). They were routinely cultured in complete Dulbecco's modified Eagle's medium (DMEM, Gibco, Grand Island, NY, USA) containing 10% foetal bovine serum (FBS, Gibco) with 100 units/ml penicillin and 100 μg/ml streptomycin (Sigma‐Aldrich, St‐Louis, MO, USA) in a humidified incubator at 37°C containing 5% CO_2_. All cell lines used were between passages three and eight for each experiment. The effect of DSF/Cu on Hep3B and HepG2 viability was determined by MTT assay. Hep3B and HepG2 cells (5 × 10^3^ cells/well) were seeded into a 96‐well plate. After overnight incubation, cells were incubated with or without the indicated concentrations of DSF/Cu for 24 hrs to test cytotoxicity.

### Transwell migration and invasion assays

Evaluation of cell migration and invasion was assessed using Transwell Permeable Supports (Corning Inc., Corning, NY, USA). Briefly, cells were allowed to grow to confluence. About 3 × 10^4^ cells/well were resuspended in 200 μl serum‐free medium with or without DSF/Cu and plated onto 8‐μm Transwell filter inserts in 24‐well plates in triplicate for migration assays, and onto Transwell filter inserts coated with Matrigel (500 μg/ml, BD Biosciences) for invasion assays. The chemoattractant in the lower chambers was 500 μl medium containing 10% foetal bovine serum, with or without different concentrations of DSF/Cu. Cells in the upper chamber were removed with a cotton swab following incubation for 24 hrs. Cells on the bottom side were fluorescently labelled with calcein‐AM. Cells were photographed by an ImageXpress‐Micro high content system (Molecular Devices, CA, USA) with a 10x dry objective lens at excitation/emission wavelengths of 530/590 nm. The images were quantified and analysed using MetaXpress software (Molecular Devices) in four random fields.

### RTCA (Real‐time cell analysis) migration and invasion assays

The assays were performed with cell invasion/migration (CIM) plates, which contain 16 modified Boyden chambers. The chambers can be used independently to measure cell migration/invasion in real time through the 8‐μm pores of a polyethylene terephthalate membrane onto gold electrodes on the underside of the membrane using the xCELLigence Analyser System (ACEA Biosciences). Experiments were set up according to the manufacturer's instructions with the membrane uncoated for migration assays and coated with Matrigel (500 μg/ml) for invasion assays. A chemotactic signal for movement was provided by inoculating 30,000–50,000 cells in serum‐free medium in the upper chamber and supplying 10% FBS in the lower chamber (with the same relevant concentration of drug). Cell index (electrical impedance) was monitored every 5 min. for the duration of the experiment. The cell index represents the capacity for cell migration or invasion, and the slope of the curve can be related to the migration velocity of tumour cells. The cell index thus reflects the migratory and invasive capacity of the tumour cells 28.

### Scratch‐wound healing recovery assays

Cells seeded in 24‐well cell culture plates were transfected at 60–70% confluency in triplicate. Growth medium was removed, and straight incisions were made with a standard 10‐μl pipette tip. Cells were washed several times with PBS to remove detached cells. Medium containing 10 ml/l FBS with or without the indicated concentrations of DSF/Cu were added to the wells and incubated for another 24 hrs. Pictures of the scratches were taken at 0 and 24 hrs. Three representative images of the scratched areas were photographed under a light microscope with 10x magnification. Images were acquired with a Leica DMI3000 B Camera System. The wound area was used to quantify the extent of wound healing in each group. The values obtained were expressed as a migration percentage, setting the gap area at 0 hr as 0%.

### Western blot analysis

About 1 × 10^7^ cells were gathered after pre‐treatment with DSF for 24 hrs and lysed in RIPA buffer (Cell Signaling Technology, Danvers, MA, USA) in the presence of protease inhibitor (PMSF) and phosphatase inhibitor (Na‐orthovanadate and NaF). Nuclear proteins were prepared using a commercial kit (Thermo Scientific, Rockford, IL, USA). Western blotting was performed as previously described 29. In brief, equal amounts of total protein extract from cultured cells or tissues were fractionated by 8–10% SDS‐PAGE and electrically transferred onto polyvinylidene difluoride (PVDF) membranes. Mouse or rabbit primary antibodies and appropriate horseradish peroxidase (HRP)‐conjugated secondary antibodies were used to detect the designated proteins. The bound secondary antibodies on the PVDF membrane were reacted with ECL detection reagents (Thermo Scientific) and exposed to X‐ray films. Results were normalized to the internal control β‐actin.

### Immunofluorescence staining

The cells were washed with PBS twice and fixed with 4% paraformaldehyde for 30 min. After permeabilization with 0.1% Triton X‐100, cells were blocked with 5% BSA for 1 hr and subsequently incubated with primary antibodies overnight. Cells were then washed with PBS and incubated with goat anti‐rabbit and antimouse IgG antibodies in the dark for 1 hr at 37°C.

### Subcutaneous tumour model

Hep3B cells (5 × 10^6^/100 μl PBS per mouse), as confirmed by trypan blue staining, were mixed with 50% Matrigel™ (BD, Bioscience, Bedford, MA, USA), and then subcutaneously (s.c.) injected into the right flank of 7‐ to 8‐week‐old male BALB/cA nude mice (Beijing Huafukang Biology Inc, Beijing, China). When the average s. c. tumour volume reached 100 mm^3^, the mice were randomly divided into four groups, which were treated with vehicle (saline only), 5‐fluorouracil (5‐FU), DSF alone, or DSF with Cu, twice a week for 29 days. Saline, 5‐FU (20 mg/kg) and DSF (60 mg/kg) were given by intravenous (i.v.) injection, and Cu (0.96 mg/kg) was given by intragastric (i.g.) administration. In the previous study 24, DSF (60 mg/kg) with Cu (9.6 mg/kg) inhibited the growth of U87‐derived tumours with a small organ index change. In this study, a lower dose of Cu was chosen to try and avoid systemic toxicity and because a low dose of Cu was effective in the *in vitro* experiments. Therefore, a DSF dose of 60 mg/kg and a Cu dose of 0.96 mg/kg were used to demonstrate the anti‐metastatic activity. This study was performed in strict accordance with the recommendations in the Guide for the Care and Use of Laboratory Animals of the National Institutes of Health. The protocol was approved by the Committee on the Ethics of Animal Experiments of the Shenyang Pharmaceutical University.

### Orthotopic liver metastasis assay

Hep3B cells were mixed with 0.02 ml PBS and slowly injected into the left hepatic lobe of the mice (4 × 10^4^ cells/mouse) after midline laparotomy. The mice were randomly divided into three groups which were treated with vehicle (saline only), DSF alone (intravenous injection, 60 mg/kg) and DSF (60 mg/kg i.v.) with or without Cu (intragastric administration, 1.9 mg/kg) twice a week for 28 days. Intrahepatic metastatic foci in hepatic lobes other than the injected lobe were determined after 28 days of treatment. This study was performed in strict accordance with the recommendations in the Guide for the Care and Use of Laboratory Animals of the National Institutes of Health. The protocol was approved by the Committee on the Ethics of Animal Experiments of the Shenyang Pharmaceutical University.

### Immunohistochemistry

Tumour samples obtained from *in vivo* studies were rinsed in PBS and fixed in 10% paraformaldehyde/PBS. Samples were dehydrated in 70% ethanol, paraffin‐embedded and sectioned (4 μm). Deparaffinized sections were stained for E‐cadherin, Vimentin, Snail+Slug, MMP2 and Smad4 antigens. Briefly, samples were rehydrated with ethanol. Tissue sections were then pre‐incubated with 10% normal goat serum in PBS (pH 7.5) followed with incubation with primary antibody overnight at 4°C. Tissue sections were then stained with biotinylated secondary antibody (Vector Laboratories, Burlingame, California, USA) for 1 hr at room temperature, followed by the Vectastain Elite ABC reagent (Vector Laboratories, Burlingame, California, USA) for 30 min. The peroxidase reaction was developed with diaminobenzidine (DAB kit; Vector Laboratories, Burlingame, California, USA), and the slides were counterstained with haematoxylin. Images were taken using a Leica DM 4000B photo microscope (magnification, ×200). Staining intensity was scored as 0 (negative), 1 (weak), 2 (medium) and 3 (strong). Extent of staining was scored as 0 (0%), 1 (1–25%), 2 (26–50%), 3 (51–75%) and 4 (76–100%), according to the percentage of the whole carcinoma area which was positively stained with each antibody. The sum of the intensity score and the extent score was used as the final staining score.

### Statistical analysis

All the data are expressed as mean values ± S.E.M. Comparisons among multiple groups were made with a one‐way analysis of variance (anova) followed by Dunnett's test. *P* < 0.05 was used for statistical significance.

## Results

### DSF/Cu potently inhibits HCC cell migration and invasion

**Figure 1:**
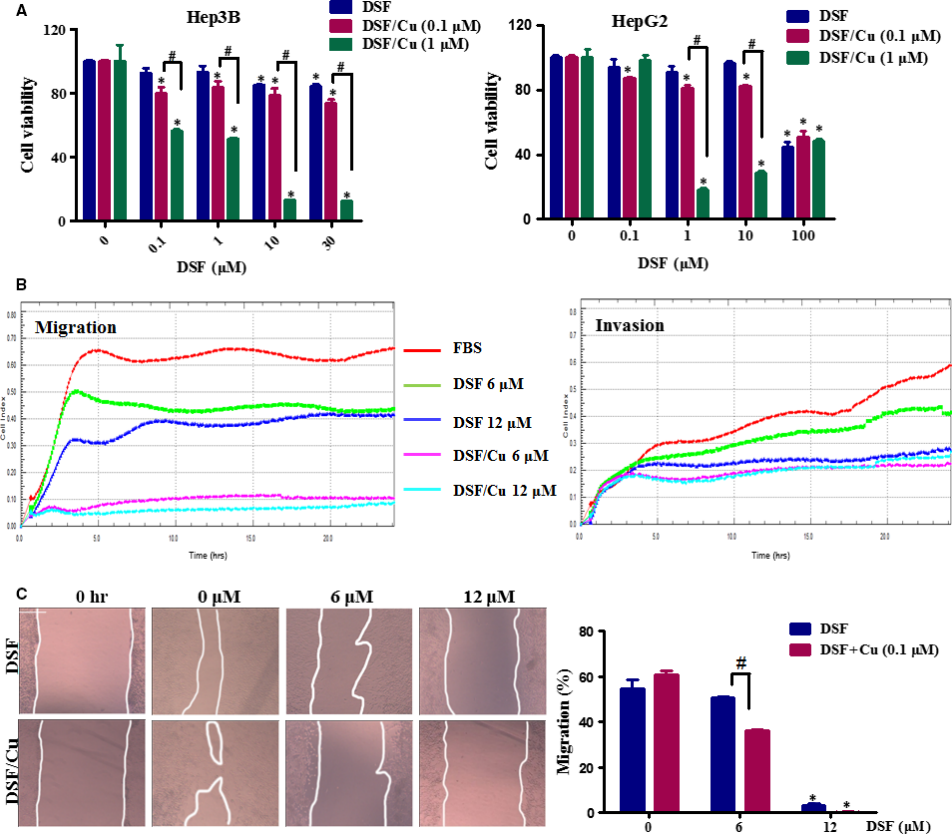
DSF/Cu inhibits the migration of Hep3B cells. (**A**) Effect of DSF/Cu on the viability of hepatic carcinoma cells. Hep3B and HepG2 cells were incubated with increasing concentrations of DSF with or without Cu (1 and 0.1 μM) for 24 hrs. Cell viability was determined by MTT colorimetric assay. The percentage of viable cells was calculated by comparison to the absorption of control cells (0.1% DMSO), which was set as 100%. The data are reported as mean ± S.E.M. of three independent experiments. *P < 0.05, significantly different compared with the DMSO‐treated control. ^#^ *P* < 0.05, *versus* the indicated groups. Data were compared by one‐way anova followed by Dunnett's test. (**B**) A Real‐time measurement of the migration and invasion of Hep3B cells during 24 hrs treatment with DSF (6, 12 μM) with or without Cu (0.1 μM). (**C**) Scratch‐wound healing recovery assay following a 24‐hrs exposure of Hep3B cells to the indicated concentrations of DSF/Cu. The wound area was used to quantify the extent of wound healing in each group. The values obtained are expressed as a migration percentage, setting the gap area at 0 hr as 0%. Scale bar, 40 μm. *P < 0.05, significantly different compared with the control group; ^#^ *P* < 0.05 *versus* the indicated groups. Comparisons were made by one‐way anova followed by Dunnett's test. The photographs were taken at the magnification of ×100.

Prior to investigating the anti‐metastatic potential of DSF/Cu, we examined the cytotoxic effect of DSF/Cu on hepatic carcinoma cells using the MTT colorimetric assay. In line with the previous report 24, our results showed that Cu greatly enhanced the inhibitory effect of DSF (shown in Fig. 1A). A significant reduction in cell viability was observed at a DSF concentration of 1 μM with Cu (1 μM) following a 24‐hrs drug exposure. The cytotoxicity was lower in the presence of Cu (0.1 μM). Based on these results, non‐cytotoxic concentrations of DSF/Cu (0.1 μM) were then used to evaluate the anti‐metastatic potential of DSF/Cu in hepatic carcinoma cells.

We used Hep3B and HepG2 cells to explore the effect of DSF/Cu on HCC cell migration. As shown in Figure 1B, real‐time cell analysis revealed that treatment with DSF inhibited the ability of Hep3B cells to migrate and invade, especially when accompanied by Cu (0.1 μM). In the scratch‐wound healing recovery assay (Fig. 1C), DSF partly inhibited wound healing of Hep3B cells, while Cu (0.1 μM) had no significant effect. Interestingly, DSF (6 and 12 μM)/Cu (0.1 μM) greatly inhibited the migration of Hep3B cells in this assay.

**Figure 2:**
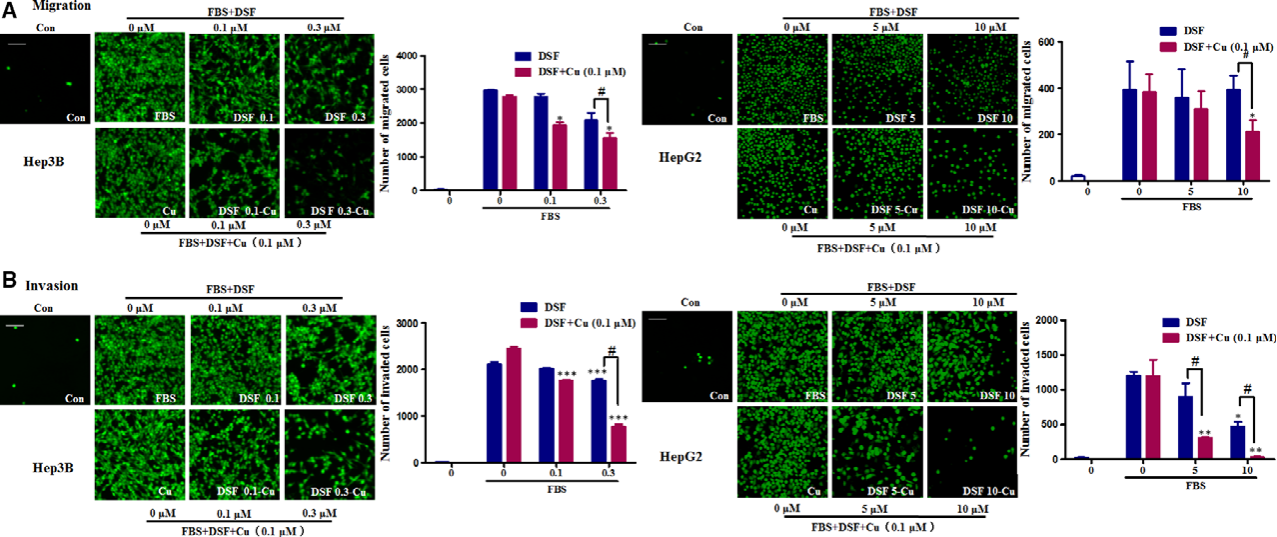
DSF/Cu inhibits the migration and invasion of HCC cells. (**A**) Representative images and quantification of the effect of DSF treatment with or without copper (II) D‐gluconate (0.1 μM) on HCC cell migration in a Transwell invasion assay. (**B**) Representative images and quantification of the effect of DSF treatment with or without copper (II) D‐gluconate (0.1 μM) on HCC cell invasion in a Transwell invasion assay. The photographs were taken at a magnification of x100 with an ImageXpress‐Micro high content system. Scale bar, 20 μm. *P < 0.05, **P < 0.01, ***P < 0.001, significantly different compared with the untreated control; ^#^ *P* < 0.05 *versus* the indicated groups. Comparisons were made by one‐way anova followed by Dunnett's test.

We then used Transwell assays to evaluate the effect of DSF/Cu on the migration and invasion ability of HCC cells. Lower concentrations of DSF were used to test whether Cu enhanced the inhibitory effect of DSF on the migration and invasion of Hep3B cells. As shown in Figure 2A, treatment with DSF alone (0.3 μM) inhibited the migration of Hep3B cells by 30%, while Cu alone (0.1 μM) did not exert any significant effect. The migration of Hep3B cells was decreased by about 48% in the presence of DSF (0.3 μM)/Cu (0.1 μM). Similarly, neither DSF (10 μM) nor Cu (0.1 μM) inhibited HepG2 cell migration, while DSF (10 μM)/Cu (0.1 μM) significantly inhibited HepG2 cell migration by 45%. The Transwell invasion assay (shown in Fig. 2B) revealed that DSF with or without Cu decreased the numbers of cells invading through Matrigel‐coated filters in a concentration‐dependent manner, and Cu dramatically improved the anti‐metastatic ability of DSF. These results indicated that Cu greatly enhanced the inhibitory effect of DSF on the migration and invasion of HCC cells.

### DSF/Cu regulates mesenchymal and epithelial phenotypes and down‐regulates NF‐κB signalling

**Figure 3:**
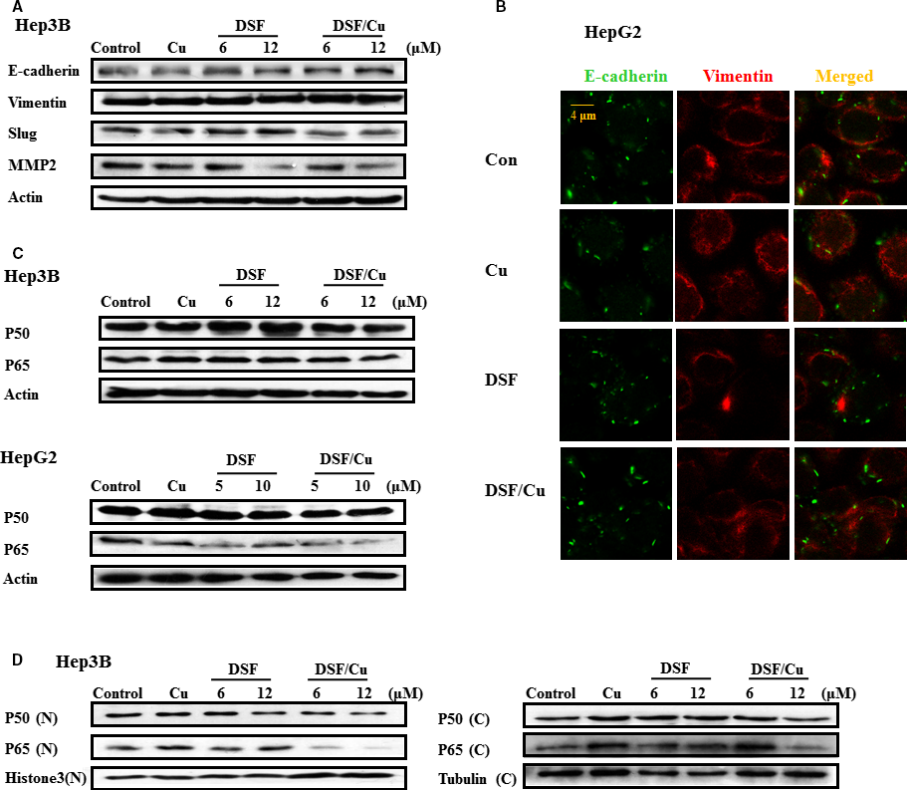
DSF/Cu regulates mesenchymal and epithelial phenotypes and NF‐κB signalling. (**A**) Protein samples were isolated from control, DSF‐treated and DSF/Cu‐treated Hep3B cells for the detection of E‐cadherin, Vimentin, Slug, MMP2 and β‐actin proteins. (**B**) The expression of E‐cadherin (green) and Vimentin (red) was examined by immunofluorescence analysis with a confocal microscope. (**C**) Protein extracts from Hep3B and HepG2 cells were immunoblotted with the specified antibodies against p65, p50 and β‐actin. (**D**) Cytoplasmic and nuclear fractions were extracted from Hep3B cells and then subjected to SDS‐PAGE/immunoblotting with anti‐NF‐κB p65 and p50 antibodies. Histone 3 and α‐tubulin were used as internal controls for cytosolic and nuclear fractions, respectively. These experiments were repeated in duplicate.

It has been reported that DSF induces E‐cadherin expression alone and in conjunction with sunitinib 22. To identify whether DSF/Cu can block the progression of EMT, analyses of mesenchymal and epithelial marker proteins were performed on HCC cells following treatment with vehicle or DSF with or without Cu (0.1 μM) for 24 hrs. Slug is a transcription factor involved in regulating EMT and cancer stem cell (CSC) phenotypes. As shown in Figure 3A, treatment with DSF/Cu (0.1 μM) for 24 hrs resulted in up‐regulation of the epithelial marker E‐cadherin and down‐regulation of Slug and the invasion‐related protein MMP2 in Hep3B cells. Immunofluorescence staining was performed in HepG2 cells. Up‐regulation of E‐cadherin was detected following a 24‐hrs treatment with DSF/Cu (shown as Fig. 3B), while Cu alone (0.1 μM) did not exert any significant effect (Fig. S1).

The NF‐κB signalling pathway appears to be a critical mediator of EMT 30. Given that DSF/Cu regulates mesenchymal and epithelial phenotypes, it is of interest to examine whether DSF/Cu suppresses NF‐κB signalling. As shown in Figure 3C, the expression of two NF‐κB subunits, p50 and p65, was unaffected by DSF/Cu. However, DSF/Cu suppressed the nuclear translocation of p50 and p65 (as shown in Fig. 3D). Therefore, we conjectured that DSF/Cu regulates mesenchymal and epithelial phenotypes through NF‐κB signalling.

### DSF/Cu represses TGF‐β1‐induced migration and EMT and regulates the expression of Smad4 in HCC cells

**Figure 4:**
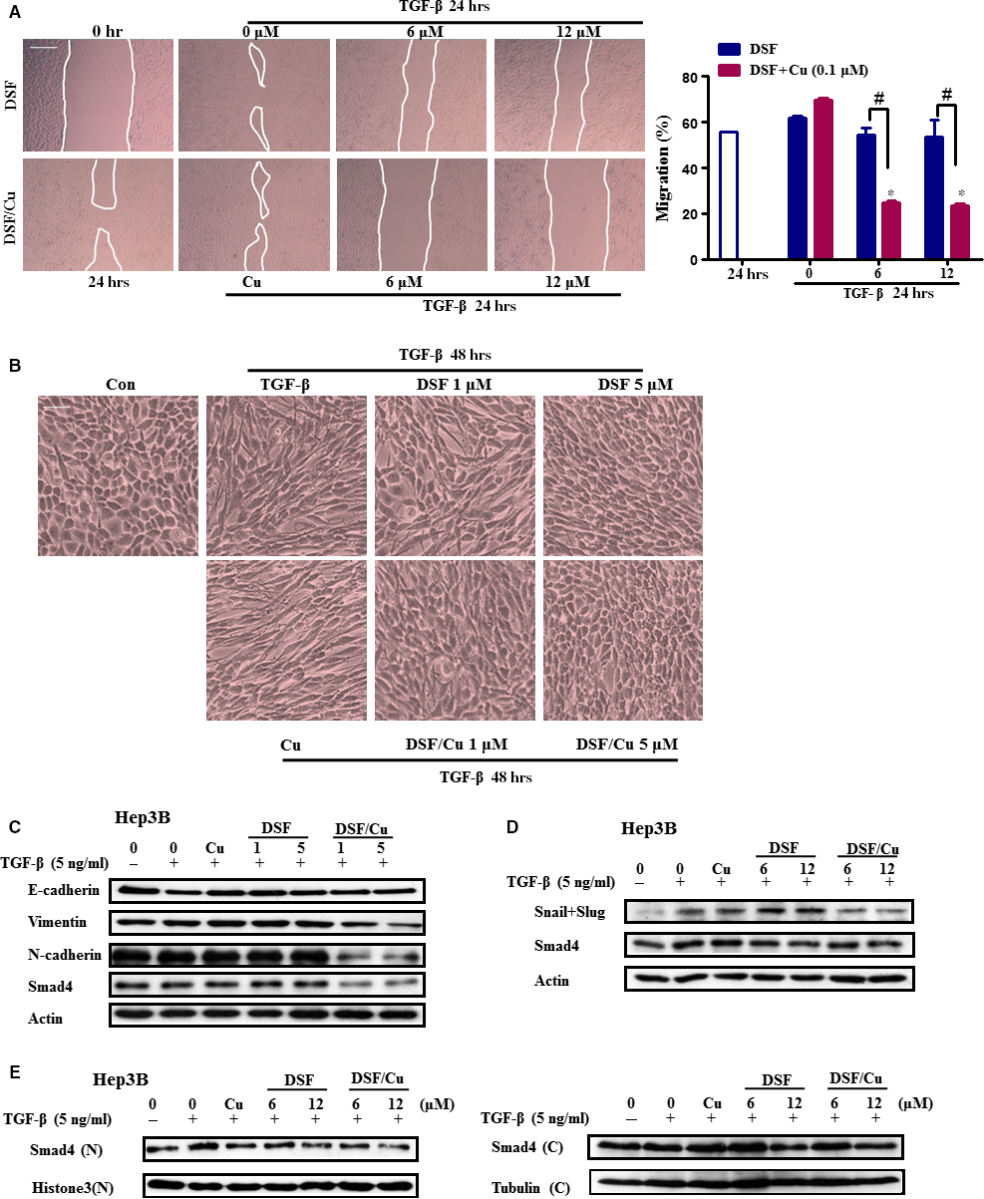
DSF/Cu represses TGF‐β1‐induced migration and regulates EMT in Hep3B cells. (**A**) Cell migration was determined by a wound healing assay. Hep3B cells were pre‐treated with DSF/Cu for 2 hrs, scratched with 10 μl pipette tips, washed to remove the debris, then covered with fresh medium containing 1% FBS and TGF‐β1 (10 ng/ml). Cells were then incubated for 24 hrs. Photographs were taken at 0 hr and 24 hrs using an inverted microscope with 100 ×  magnification. The wound area was used to quantify the extent of wound healing in each group. The values obtained are expressed as a migration percentage, setting the gap area at 0 hr as 0%. Scale bar, 40 μm. *P < 0.05, significantly different compared with the control group; ^#^ *P* < 0.05 *versus* the indicated groups. (**B–C**) Cells were pre‐treated with DSF/Cu for 2 hrs and then stimulated with TGF‐β1 for 48 hrs. (**B**) Morphological changes in Hep3B cells after exposure to DSF with or without Cu. Images were taken under a light microscope (× 200 magnification). Scale bar, 10 μm. (**C**) Protein samples were isolated from control and TGF‐β‐treated cells with or without DSF/Cu for the detection of E‐cadherin, Vimentin, N‐cadherin and β‐actin. (**D**) Cells were pre‐treated with DSF/Cu for 24 hrs and then stimulated with TGF‐β1 for 6 hrs for the detection of Snail, Smad4 and β‐actin. (**E**) Cytoplasmic fractions and nuclear fractions were extracted and then subjected to SDS‐PAGE/immunoblotting with anti‐Smad4, Histone 3 and α‐tubulin antibodies.

TGF‐β1 is reported to be a major secretory ligand that stimulates Smad2/Smad3 activation by acting through the TGF‐β‐type I receptor (TβRI). The activated Smad complex binds to Smad binding elements (SBEs) in DNA, leading to transcription of several EMT regulatory genes, including Snail and Slug 31, 32. TGF‐β1 treatment may cause EMT through the up‐regulation of transcriptional factors that regulate EMT in HCC cells 33. As shown in Figure 4A, TGF‐β1 increased the migration of Hep3B cells, and this increase was almost completely blocked by DSF/Cu, and attenuated by DSF (Fig. 4A). Morphological changes which are indicative of EMT were also detected. When Hep3B cells were treated with TGF‐β, they acquired a fibroblast‐like, mesenchymal morphology (Fig. 4B). After treatment with increasing concentrations of DSF/Cu, these cells kept a more epithelial‐like appearance even after stimulation by TGF‐β (Fig. 4B). Western blot analysis showed that pre‐treatment with DSF/Cu other than DSF alone inhibited the TGF‐β1‐induced expression of the mesenchymal markers Vimentin and N‐cadherin, as well as Smad4 (Fig. 4C). Pre‐treatment of Hep3B cells for 24 hr with DSF/Cu also inhibited the expression of Snail (Fig. 4D). The regulation of the mesenchymal marker by DSF/Cu was also confirmed in HepG2 cell line (Fig. S2). Furthermore, DSF/Cu suppressed the nuclear translocation of Smad4 induced by TGF‐β1 (Fig. 4E). These results revealed that TGF‐β signalling played an important role in the anti‐metastatic effects of DSF/Cu.

### DSF/Cu regulates the metastatic ability of Hep3B cells through the NF‐κB pathway

**Figure 5:**
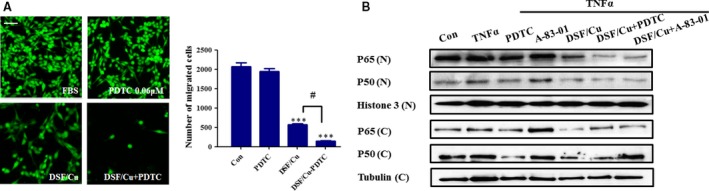
DSF/Cu regulates the metastatic ability of Hep3B cells through the NF‐κB pathway. (**A**) Representative images showing the effect of a 24‐hrs exposure to PDTC (NF‐κB signalling inhibitor), DSF/Cu and PDTC plus DSF/Cu on Hep3B cells in a Transwell migration assay, together with quantification of the number of migrated cells. The photographs were taken at a magnification of x100 with an ImageXpress‐Micro high content system. Scale bar, 20 μm. ***P < 0.001, significantly different compared with the untreated control. ^#^ *P* < 0.05 *versus* the indicated groups. (**B**) Hep3B cells were stimulated with TNFα for 6 hrs. The cytoplasmic fractions (**C**) and nuclear fractions (*N*) were extracted and then subjected to SDS‐PAGE/immunoblotting with anti‐NF‐κB p65, anti‐NF‐κB p50, Histone 3 and α‐tubulin antibodies. Histone 3 and α‐tubulin served as internal controls for the nuclear and cytosolic fractions, respectively.

The NF‐κB pathway is an important regulator of epidermal homeostasis, inflammatory responses and carcinogenesis. To confirm the role of NF‐κB signalling, we used a specific inhibitor of the NF‐κB pathway (PDTC, 0.06 μM). The concentration of PDTC was chosen to slightly inhibit the migration of Hep3B cells. As shown in Figure 5A, PDTC significantly enhanced the inhibitory effect of DSF/Cu on Hep3B migration. As shown in Figure 5B, DSF/Cu also inhibited the TNFα‐induced nuclear translocation of p50 and p65, and the inhibitory effect was enhanced by PDTC. A previous study suggested that the TGF‐β was required for NF‐κB‐dependent gene expression 34. Earlier work has shown that the extensive crosstalk between these pathways depends on the cell type and context. The TNFα‐induced nuclear translocation of p50 and p65 was not inhibited by A‐83‐01 (an inhibitor of the TGF‐β pathway) in Hep3B cells (Fig. 5B). Therefore, we suggest that the correlation between the TGF‐β1 and NF‐κB pathways in the present study was not as close as previously reported.

### DSF/Cu inhibits lung metastasis of Hep3B cells in BALB/c nude mice *in vivo*

**Figure 6:**
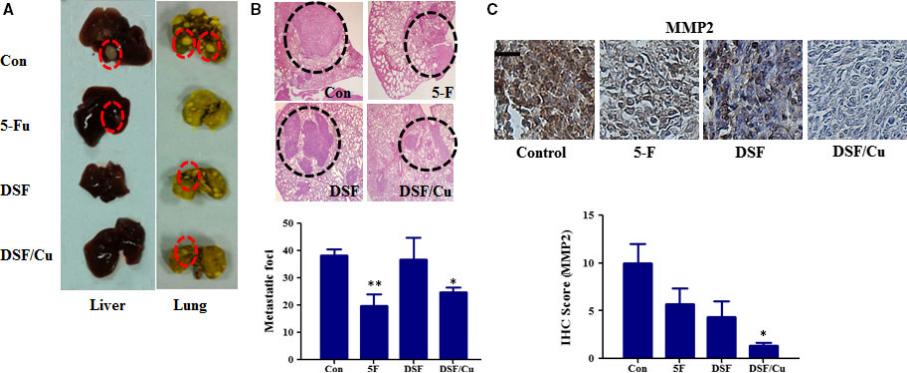
*In vivo* anti‐metastatic activities of DSF/Cu in mice subcutaneously implanted with Hep3B cells. Mice with subcutaneous xenografts of human Hep3B cells were randomly divided into four groups (*n* = 8 per group) and given injections of saline (vehicle control), 5‐Fu or DSF (60 mg/kg, i.v.) with or without Cu (0.96 mg/kg, i.g.) twice a week for 29 days. The tumours were then removed for analysis. (**A**) Liver and lung metastasis was observed. Only a few metastases (red dashed circles) were found in mice treated with DSF/Cu. (**B**) Representative haematoxylin and eosin (HE) staining confirmed the development of tumours (black dashed circles) in lung tissue. The lower panel shows the number of metastatic nodules that were counted on the lung surface. (**C**) Immunohistochemical staining and quantification of MMP2. Scale bar, 20 μm. *P < 0.05, significantly different compared with the vehicle‐only control group; **P < 0.01 compared with control group.

To further investigate whether Cu facilitates the anti‐metastatic activity of DSF *in vivo*, a Hep3B subcutaneous tumour model was established in BALB/c nude mice. DSF (60 mg/kg) was administered by intravenous injection with or without Cu (intragastric administration, 0.96 mg/kg) twice a week for 29 days. To further investigate the effect of DSF/Cu on Hep3B metastasis *in vivo*, the mice were killed, their lungs were collected, and the number of nodules on the surface was counted. As shown in Figure 6A and B, histological analysis demonstrated that intrahepatic tumour nodules were larger in control mice than in mice treated with DSF/Cu. Pathological analysis demonstrated that the metastatic tumour nodules in control mice were larger and more numerous than those in DSF/Cu‐treated mice. The mean number of metastatic nodules on the surface of the lungs in the control group was 38.33. The mean number of metastatic nodules in the mice treated with DSF/Cu was 24.67, which was significantly lower than in the control group. The pulmonary tumour nodules were then evaluated for the expression of MMP2 (Fig. 6C). Consistent with the *in vitro* results, the expression of MMP2 in the DSF/Cu‐treated mice was lower than that in the control mice.

**Figure 7:**
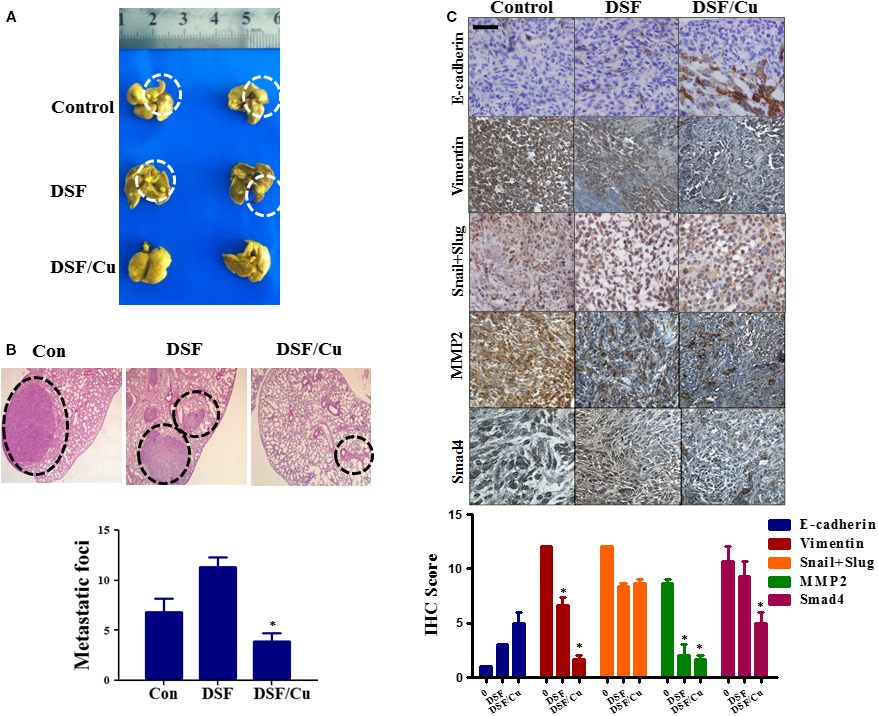
*In vivo* anti‐metastatic activities of DSF/Cu in a Hep3B orthotopic mouse model. Mice with Hep3B human orthotopic xenografts were randomly divided into three groups (*n* = 8 per group) and given injections of vehicle (saline only), and DSF (60 mg/kg, i.v.) with or without Cu (1.9 mg/kg, i.g.). (**A**) Metastases (white dashed circles) were observed in the lungs of mice. Only a few metastases were found in mice treated with DSF/Cu. (**B**) Representative haematoxylin and eosin (HE) staining of lung sections, showing metastatic tumours (black dashed circles). The graph shows quantification of metastatic nodules on the surface of the lungs. *P < 0.05, significantly different compared with the control group. (**C**) Immunohistochemical staining and quantification of E‐cadherin, Vimentin, Slug, MMP2 and Smad4 in Hep3B orthotopic xenograft tumours. The photographs were taken at magnifications of ×200. Scale bar, 20 μm. *P < 0.05, significantly different compared with the control group.

We also examined the *in vivo* anti‐tumour effects of DSF/Cu in an orthotopic mouse model of liver cancer using Hep3B cells. The orthotopic xenograft model is superior to the subcutaneous xenograft model in terms of replicating the tumour microenvironment 35. The model was established by injecting Hep3B cells into the liver of nude mice. After 4 weeks, mice were killed and their lungs were collected. As shown in Figure 7A and B, pathological analysis demonstrated that the metastatic tumour nodules were fewer and smaller in size in the DSF/Cu‐treated mice than in the control mice. The mean number of metastatic nodules on the surface of the lungs in the control group was 6.8. The mean number of metastatic nodules in the DSF/Cu‐treated mice was 3.9, which was significantly lower than in the control group. The intrahepatic tumour nodules were then evaluated for expression of E‐cadherin, Vimentin, Snail+Slug, MMP2 and Smad4 by immunohistochemical analysis. As shown in Figure 7C, the expression of Vimentin, MMP2 and Smad4 was more effectively down‐regulated by DSF/Cu than by DSF alone, and the expression of E‐cadherin was up‐regulated. Taken together, our results illustrated that DSF/Cu inhibits the metastasis of hepatocellular carcinoma both *in vitro* and *in vivo* and regulates EMT, which could be induced by TGF‐β and NF‐κB pathway.

## Discussion

HCC is a common malignancy in many countries. The low survival rate of late‐stage HCC is largely due to the high rate of intrahepatic and extrahepatic metastasis 36. Recently, there has been increasing recognition that discovering new therapeutic uses for ‘old’ drugs is a valuable approach, because these drugs are well tolerated in the human body with fewer toxic effects. DSF, a Food and Drug Administration‐approved anti‐alcoholism drug, has been used in the clinic, and extensive pre‐clinical and clinical data are available 37.

Cu plays an important role in inflammation and the growth of tumours 38. The concept of using Cu to tackle cancer was proposed many decades ago 39. Cu can stimulate the proliferation and migration of endothelial cells at high concentrations 40. DSF was demonstrated to be a potent proteasome inhibitor and apoptosis inducer *in vitro* and *in vivo* only when Cu was present 41. However, the anti‐metastatic ability of DSF/Cu in HCC remained unknown. The present research used Hep3B and HepG2 cells to investigate the anti‐metastatic effect of DSF/Cu. Consistent with a previous report 21, our study showed that DSF/Cu inhibited the proliferation, migration and invasion of these two cell lines. Previous studies also demonstrated that DSF strongly suppressed the lung metastasis of human fibrosarcoma cells in nude mice with no significant effects on the volume of the primary tumours 42. Therefore, we further examined the role of DSF/Cu in suppressing tumour metastasis using subcutaneous and orthotopic mouse models. We found that DSF/Cu inhibited hepatocellular carcinoma metastasis more effectively than DSF *in vitro* and *in vivo*. It was also reported that DSF suppressed the invasive ability of osteosarcoma 21, and the high metastatic potential of human lung adenocarcinoma, human bladder adenocarcinoma 20 and breast cancer 25. Therefore, we conjecture that DSF/Cu will also regulate the metastatic potential of other carcinomas, a hypothesis which remains to be investigated in the future.

Epithelial–mesenchymal transition (EMT) is an important program in cancer metastasis. It is characterized by loss of epithelial cell polarity and acquisition of elongated mesenchymal morphology, concomitant with disruption of cell adhesion, increased cell migration, invasion and metastasis and chemotherapeutic resistance 43, 44. Although multiple pathways participate in EMT, the best studied factors are NF‐κB and TGF‐β 45, 46, 47. EMT was verified to be involved in the cascade of signalling events inducing HCC metastasis 48, 49, 50, 51. TGF‐β promotes EMT through a combination of SMAD‐dependent and SMAD‐independent pathways 52, 53. In line with previous reports 22, 25, our results showed that DSF induced E‐cadherin expression. Moreover, DSF/Cu reversed EMT more effectively than DSF alone. Importantly, DSF/Cu regulated the expression of Smad4, which plays an important role in the EMT program.

NF‐κB activation plays a major role in EMT 54. NF‐κB induces and maintains EMT in model systems through two mechanisms, the up‐regulation of EMT master‐switch transcription factors 55, 56 and stabilization of Snail 57. Snail is one of the NF‐κB target genes and suppresses the expression of adherens junction proteins 58. Furthermore, it has also been reported that the inhibition of NF‐κB abrogates TGF‐β1 stimulation of EMT, cell motility and invasion and that HCC metastasis can be inhibited by repressing p65/NF‐κB activation both *in vitro* and *in vivo* 59. Coordinated activation of TNF and TGF‐β signalling cascades effectively induces EMT and the expression of genes related to dedifferentiation and stemness 60. Therefore, we focused on TGF‐β and NF‐κB signalling. The results showed that DSF/Cu inhibited the nuclear translocation of the NF‐κB subunits p50 and p65. The NF‐κB‐selective inhibitor PDTC was also used to confirm the role of the NF‐κB pathway in the regulation of migration induced by DSF/Cu. The metastatic ability of HCC cells was reduced by inhibition of NF‐κB signalling and was further decreased by the combination of DSF/Cu and PDTC. When Hep3B cells were treated with DSF/Cu, the expression and nuclear translocation of Smad4 were reduced. It was reported that binding of TGF‐β family ligands to their receptors leads to subsequent phosphorylation of Smad2/3, which are then translocated into the nucleus after forming a complex with Smad4 to regulate the expression of transcription factors implicated in initiating the EMT program, including Snail, Twist, Zeb1 and Slug 61. Multiple studies have indicated that Smad4 has oncogenic functions in several kinds of human cancer, and overexpression of Smad4 was observed in the tumours of HCC patients compared with adjacent tissues 62. Knockdown of Smad4 significantly reduced the colony formation and migratory capacity of HCC cells and inhibited cell migration, and invasion in gemcitabine‐resistant (GR) HCC cells 63. E‐cadherin, a key player in EMT, is a target gene in the SMAD4 signalling network 64. The TGF‐β‐activated Smad3/4 complex may also directly interact with specific SBEs in the *N‐cadherin* promoter 65. Therefore, we suggest that the inhibition of Smad4 and TGF‐β signalling contributes to the DSF/Cu‐induced anti‐metastatic effect and the down‐regulation of EMT in hepatocellular carcinoma cells.

The TGF‐β and NF‐κB pathways are important regulators of epidermal homeostasis, inflammatory responses and carcinogenesis. It was reported that TGF‐β could induce IκBα phosphorylation followed by translocation of the NF‐κB p65 subunit into the nucleus and increased NF‐κB activity 66. Previous studies have also shown that extensive crosstalk between these pathways is dependent on cell type and context 34. We found that A‐83‐01 did not inhibit the TNFα‐induced translocation of p50 and p65. Therefore, we suggest that DSF/Cu inhibits the TGF‐β1 and NF‐κB pathways individually to regulate EMT and the migration of HCC cells.

## Conclusion

DSF/Cu exerted an inhibitory effect on EMT in HCC cells, which corresponded to observed decreases in migration and invasiveness *in vitro* as well as reduced experimental metastasis *in vivo*. The NF‐κB pathway and Smad signalling were found to be key players in the DSF/Cu‐induced suppression of HCC EMT. These pre‐clinical findings may provide a plausible scientific basis for clinical research into the ability of DSF/Cu to reduce the invasiveness of HCC.

## Conflict of interest

There is no conflict of interest declared by the authors.

## Supporting information


**Figure S1** The quantification of expression of E‐cadherin and Vimentin in HepG2 cells by immunofluorescence analysis with a confocal microscope.

**Figure S2** Western blotting test of E‐cadherin, Vimentin, and N‐cadherin.
