# Supplementary figures and images for: Disulfiram combined with copper inhibits metastasis and epithelial–mesenchymal transition in hepatocellular carcinoma through the NF‐κB and TGF‐β pathways
Source: J Cell Mol Med. 2017 Nov 17;22(1):439–51. doi: 10.1111/jcmm.13334 (PMC5742719; doi:10.1111/jcmm.13334)

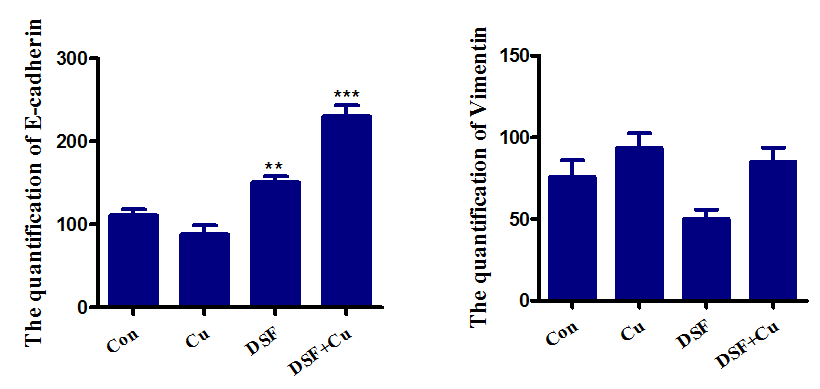

Supplement: Supplementary file 1 — Figure S1 The quantification of expression of E‐cadherin and Vimentin in HepG2 cells by immunofluorescence analysis with a confocal microscope. [file JCMM-22-439-s001.tiff]

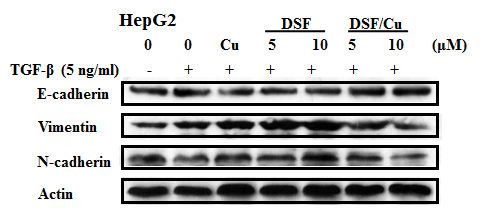

Supplement: Supplementary file 2 — Figure S2 Western blotting test of E‐cadherin, Vimentin, and N‐cadherin. [file JCMM-22-439-s002.tiff]
